# Ethyl 1-(2-hy­droxy­eth­yl)-2-*p*-tolyl-1*H*-benzimidazole-5-carboxyl­ate

**DOI:** 10.1107/S1600536810020799

**Published:** 2010-06-05

**Authors:** Natarajan Arumugam, Aisyah Saad Abdul Rahim, Habibah A Wahab, Jia Hao Goh, Hoong-Kun Fun

**Affiliations:** a School of Pharmaceutical Sciences, Universiti Sains Malaysia, 11800 USM, Penang, Malaysia; b Division of Advanced Drug Delivery, Malaysian Institute of Pharmaceuticals and Nutraceuticals, Malaysia; c X-ray Crystallography Unit, School of Physics, Universiti Sains Malaysia, 11800 USM, Penang, Malaysia

## Abstract

The asymmetric unit of the title compound, C_19_H_20_N_2_O_3_, contains two mol­ecules (*A* and *B*) with slightly different orientations of the ethyl groups with respect to the attached carboxyl­ate groups. Intra­molecular C—H⋯O hydrogen bonds generate *S*(8) ring motifs in both mol­ecules *A* and *B*. In each mol­ecule, the benzimidazole ring system is essentially planar, with maximum deviations of 0.023 (1) and 0.020 (1) Å, respectively, for mol­ecules *A* and *B*. The dihedral angle between the benzimidazole ring system and the phenyl ring is 37.34 (5)° for mol­ecule *A* and 42.42 (5)° for mol­ecule *B*. In the crystal, O—H⋯N and C—H⋯O hydrogen bonds link the mol­ecules into [100] columns with a cross-section of two-mol­ecule by two-mol­ecule wide, and further stabilization is provided by weak C—H⋯π and π–π inter­actions [centroid separations = 3.5207 (7) and 3.6314 (8) Å].

## Related literature

For general background to and applications of benzimidazole derivatives, see: Denny *et al.* (1990 ▶); Evans *et al.* (1997 ▶); Grassmann *et al.* (2002 ▶); Göker *et al.* (2002 ▶); Seth *et al.* (2003 ▶). For graph-set descriptions of hydrogen-bond ring motifs, see: Bernstein *et al.* (1995 ▶). For closely related benzimidazole structures, see: Arumugam *et al.* (2010**a* ▶,*b* ▶,c* ▶). For the stability of the temperature controller used for the data collection, see: Cosier & Glazer (1986 ▶).
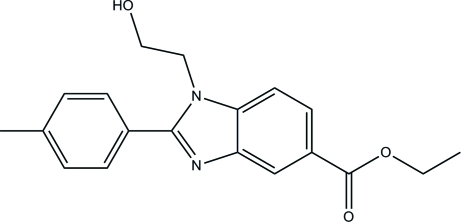


## Experimental

### 

#### Crystal data

C_19_H_20_N_2_O_3_
*M* *_r_* = 324.37
Triclinic, 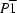

*a* = 9.0400 (9) Å
*b* = 12.6806 (13) Å
*c* = 15.5504 (17) Å
α = 74.170 (2)°
β = 74.360 (2)°
γ = 76.721 (2)°
*V* = 1627.9 (3) Å^3^
*Z* = 4
Mo *K*α radiation
μ = 0.09 mm^−1^
*T* = 100 K
0.41 × 0.32 × 0.23 mm

#### Data collection

Bruker APEXII DUO CCD diffractometer
Absorption correction: multi-scan (*SADABS*; Bruker, 2009 ▶) *T*_min_ = 0.964, *T*_max_ = 0.980
30776 measured reflections
10646 independent reflections
8773 reflections with *I* > 2σ(*I*)
*R*_int_ = 0.030

#### Refinement

*R*[*F* ^2^ > 2σ(*F* ^2^)] = 0.048
*wR*(*F* ^2^) = 0.194
*S* = 1.13
10646 reflections
445 parameters
H atoms treated by a mixture of independent and constrained refinement
Δρ_max_ = 0.60 e Å^−3^
Δρ_min_ = −0.65 e Å^−3^

### 

Data collection: *APEX2* (Bruker, 2009 ▶); cell refinement: *SAINT* (Bruker, 2009 ▶); data reduction: *SAINT*; program(s) used to solve structure: *SHELXTL* (Sheldrick, 2008 ▶); program(s) used to refine structure: *SHELXTL*; molecular graphics: *SHELXTL*; software used to prepare material for publication: *SHELXTL* and *PLATON* (Spek, 2009 ▶).

## Supplementary Material

Crystal structure: contains datablocks global, I. DOI: 10.1107/S1600536810020799/hb5474sup1.cif

Structure factors: contains datablocks I. DOI: 10.1107/S1600536810020799/hb5474Isup2.hkl

Additional supplementary materials:  crystallographic information; 3D view; checkCIF report

## Figures and Tables

**Table 1:** Hydrogen-bond geometry (Å, °) *Cg*1 is the centroid of the N1*B*/C7*B*/N2*B*/C8*B*/C13*B* 4,5-dihydro imidazole ring.

*D*—H⋯*A*	*D*—H	H⋯*A*	*D*⋯*A*	*D*—H⋯*A*
O3*A*—H1*OA*⋯N1*B*	0.96 (3)	1.83 (3)	2.7847 (14)	173 (2)
O3*B*—H1*OB*⋯N1*A*	0.81 (2)	2.08 (2)	2.8859 (15)	172 (3)
C1*A*—H1*AA*⋯O3*A*	0.93	2.37	3.2473 (16)	156
C1*B*—H1*BA*⋯O3*B*	0.93	2.36	3.2331 (16)	157
C12*B*—H12*B*⋯O3*A*^i^	0.93	2.45	3.2788 (16)	149
C15*B*—H15*C*⋯O1*B*^ii^	0.97	2.55	3.2889 (17)	133
C18*A*—H18*A*⋯O1*A*^iii^	0.97	2.58	3.1928 (16)	121
C17*B*—H17*C*⋯*Cg*1^iv^	0.97	2.70	3.4194 (13)	131

Symmetry codes: (i) 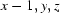; (ii) 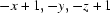; (iii) 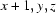; (iv) 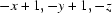.

**Fig. 1.**
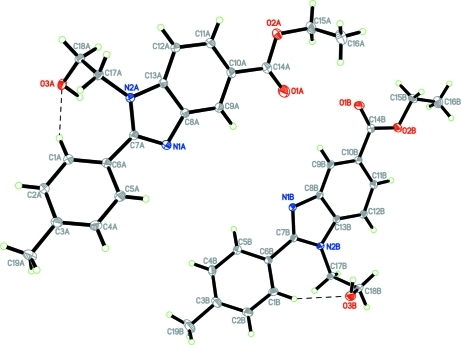
The molecular structure of (I) with 50% probability displacement ellipsoids for non-H atoms. Intramolecular hydrogen bonds are shown as dashed lines.

**Fig. 2.**
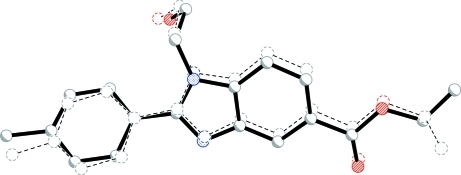
Fit of molecule A (dashed lines) on molecule B (solid lines). H atoms have been omitted for clarity.

**Fig. 3.**
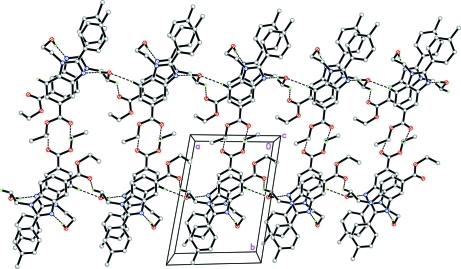
The crystal packing of (I), viewed down c axis, showing a two-molecule by two-molecule wide column along the a axis. H atoms not involved in intermolecular interactions (dashed lines) have been omitted for clarity.

## supplementary crystallographic information

### Comment

Benzimidazoles are known to exhibit anti-HIV (Evans *et al.*, 1997), anti-fungal (Göker *et al.*, 2002) and anti-parasitic (Seth *et al.*, 2003) activities. In particular, substituted benzimidazoles have proven as drug leads, generating pharmacological interests (Grassmann *et al.*, 2002). A series of substituted benzimidazole derivatives have been synthesised and evaluated for *in vitro* and *in vivo* anti-tumor activity and DNA binding affinity (Denny *et al.*, 1990). Due to their importance, the crystal structure determination of the title compound was carried out and the results are presented in this paper.

The asymmetric unit of the title benzimidazole compound (Fig. 1) comprises of two crystallographically independent molecules, designated *A* and *B*. A superposition of the non-H atoms of molecules *A* and *B* (Fig. 2) using *XP* in *SHELXTL* (Sheldrick, 2008), gave an r.m.s. deviation of 0.517 Å. Molecules *A* and *B* differ slightly in the orientation of the ethyl groups (C15 and C16) with respect to the attached carboxylate groups, as can be seen in Fig. 2. The torsion angles C14A–O2A–C15A–C16A and C14B–O2B–C15B–C16B are -77.41 (15) and -171.37 (10)°, respectively.

Intramolecular C1A—H1AA···O3A and C1B—H1BA···O3B hydrogen bonds generate eight-membered rings, producing *S*(8) ring motifs (Bernstein, 1995). In each molecule, the benzimidazole ring system (C7-C13/N1/N2) are essentially planar, with maximum deviations of 0.023 (1) and -0.020 (1) Å, respectively, at atoms C7A of molecule *A* and C8B of molecule *B*. In molecule *A*, the benzimidazole ring system is inclined at dihedral angle of 37.34 (5)° with the C1A-C6A phenyl ring; the respective angle for molecule *B* is 42.42 (5)°. The geometric parameters are comparable to those reported in closely related benzimidazole structures (Arumugam *et al.*, 2010*a,b,c*).

In the crystal packing (Fig. 3), intermolecular O3A—H1OA···N1B, O3B—H1OB···N1A, C12B—H12B···O3A, C15B—H15C···O1B and C18A—H18A···O1A hydrogen bonds link the molecules into columns with cross-section of two-molecule by two-molecule wide along the *a* axis. The crystal packing is further stabilized by intermolecular C17B—H17C···*Cg*1 interactions (Table 1) as well as weak π–π aromatic stacking interactions [*Cg*1···*Cg*2 = 3.5207 (7) and *Cg*3···*Cg*4 = 3.6314 (8) Å; symmetry code: x, y, z; *Cg*1 and *Cg*2 are the centroids of 4,5-dihydroimidazole rings (C7A/N1A/C8A/C13A/N2A and C7B/N1B/C8B/C13B/N2B), respectively; *Cg*3 and *Cg*4 are the centroids of C8A-C13A and C8B-C13B phenyl rings, respectively].

### Experimental

A solution of ethyl-3-amino-4-(2-hydroxyethylamino) benzoate (0.5 g, 2.22 mmol) and sodium bisulfite adduct of *p*-methyl benzaldehyde (1.0 g, 4.46 mmol) in DMF was treated under microwave conditions at 403 K. The reaction mixture was then diluted in EtOAc (30 ml) and washed with H_2_O (30 ml). The organic layer was collected and dried over Na_2_SO_4_. The solvent was removed under reduced pressure to afford the crude product, which upon recrystallisation from EtOAc, revealed the title compound as colourless crystals.

### Refinement

Hydroxy H-atoms were located from the difference Fourier map and allowed to refine freely. The remaining H atoms were place in their calculated positions, with C–H = 0.93–0.97 Å and *U*_iso_(H) = 1.2 or 1.5 *U*_eq_(C). The rotating group model was applied for the methyl groups.  The highest residual electron density peak is 0.49 Å from N2B and the deepest hole is 0.85 Å from C8B.

### Crystal data

**Table:**

C_19_H_20_N_2_O_3_	*Z* = 4
*M**_r_* = 324.37	*F*(000) = 688
Triclinic, *P*1	*D*_x_ = 1.324 Mg m^−^^3^
Hall symbol: -P 1	Mo *K*α radiation, λ = 0.71073 Å
*a* = 9.0400 (9) Å	Cell parameters from 9690 reflections
*b* = 12.6806 (13) Å	θ = 2.8–33.8°
*c* = 15.5504 (17) Å	µ = 0.09 mm^−^^1^
α = 74.170 (2)°	*T* = 100 K
β = 74.360 (2)°	Block, colourless
γ = 76.721 (2)°	0.41 × 0.32 × 0.23 mm
*V* = 1627.9 (3) Å^3^	

### Data collection

**Table:**

Bruker APEXII DUO CCD diffractometer	10646 independent reflections
Radiation source: fine-focus sealed tube	8773 reflections with *I* > 2σ(*I*)
graphite	*R*_int_ = 0.030
φ and ω scans	θ_max_ = 31.5°, θ_min_ = 2.4°
Absorption correction: multi-scan (*SADABS*; Bruker, 2009)	*h* = −13→13
*T*_min_ = 0.964, *T*_max_ = 0.980	*k* = −18→18
30776 measured reflections	*l* = −22→22

### Refinement

**Table:**

Refinement on *F*^2^	Primary atom site location: structure-invariant direct methods
Least-squares matrix: full	Secondary atom site location: difference Fourier map
*R*[*F*^2^ > 2σ(*F*^2^)] = 0.048	Hydrogen site location: inferred from neighbouring sites
*wR*(*F*^2^) = 0.194	H atoms treated by a mixture of independent and constrained refinement
*S* = 1.13	*w* = 1/[σ^2^(*F*_o_^2^) + (0.1301*P*)^2^ + 0.1152*P*] where *P* = (*F*_o_^2^ + 2*F*_c_^2^)/3
10646 reflections	(Δ/σ)_max_ < 0.001
445 parameters	Δρ_max_ = 0.60 e Å^−^^3^
0 restraints	Δρ_min_ = −0.65 e Å^−^^3^

### Special details

**Table:**

Experimental. The crystal was placed in the cold stream of an Oxford Cryosystems Cobra open-flow nitrogen cryostat (Cosier & Glazer, 1986) operating at 100.0 (1)K.
Geometry. All esds (except the esd in the dihedral angle between two l.s. planes) are estimated using the full covariance matrix. The cell esds are taken into account individually in the estimation of esds in distances, angles and torsion angles; correlations between esds in cell parameters are only used when they are defined by crystal symmetry. An approximate (isotropic) treatment of cell esds is used for estimating esds involving l.s. planes.
Refinement. Refinement of F^2^ against ALL reflections. The weighted R-factor wR and goodness of fit S are based on F^2^, conventional R-factors R are based on F, with F set to zero for negative F^2^. The threshold expression of F^2^ > 2sigma(F^2^) is used only for calculating R-factors(gt) etc. and is not relevant to the choice of reflections for refinement. R-factors based on F^2^ are statistically about twice as large as those based on F, and R- factors based on ALL data will be even larger.

### Fractional atomic coordinates and isotropic or equivalent isotropic displacement parameters (Å^2^)

**Table:**

	*x*	*y*	*z*	*U*_iso_*/*U*_eq_	
O1A	0.07593 (12)	0.34205 (9)	0.46417 (7)	0.0276 (2)	
O2A	0.22621 (11)	0.21688 (8)	0.55131 (7)	0.0214 (2)	
O3A	0.96752 (10)	0.50727 (7)	0.18794 (6)	0.01475 (17)	
N1A	0.44922 (12)	0.63318 (8)	0.29752 (7)	0.01358 (19)	
N2A	0.68064 (11)	0.55873 (8)	0.33553 (6)	0.01221 (18)	
C1A	0.79520 (14)	0.76316 (10)	0.18103 (8)	0.0153 (2)	
H1AA	0.8706	0.6999	0.1772	0.018*	
C2A	0.83043 (14)	0.86714 (10)	0.13230 (8)	0.0170 (2)	
H2AA	0.9302	0.8723	0.0965	0.020*	
C3A	0.72088 (14)	0.96366 (10)	0.13554 (8)	0.0164 (2)	
C4A	0.57258 (15)	0.95372 (10)	0.19112 (8)	0.0172 (2)	
H4AA	0.4977	1.0172	0.1953	0.021*	
C5A	0.53556 (14)	0.85052 (10)	0.24015 (8)	0.0161 (2)	
H5AA	0.4360	0.8456	0.2763	0.019*	
C6A	0.64640 (13)	0.75385 (9)	0.23574 (8)	0.0132 (2)	
C7A	0.59444 (13)	0.64791 (9)	0.28754 (7)	0.0127 (2)	
C8A	0.43877 (13)	0.52957 (9)	0.35614 (7)	0.0123 (2)	
C9A	0.31274 (13)	0.47294 (9)	0.39126 (8)	0.0139 (2)	
H9AA	0.2176	0.5029	0.3748	0.017*	
C10A	0.33376 (14)	0.37013 (9)	0.45175 (8)	0.0141 (2)	
C11A	0.47717 (14)	0.32492 (10)	0.47744 (8)	0.0148 (2)	
H11A	0.4869	0.2567	0.5191	0.018*	
C12A	0.60436 (14)	0.37979 (9)	0.44210 (8)	0.0141 (2)	
H12A	0.6996	0.3498	0.4584	0.017*	
C13A	0.58203 (13)	0.48226 (9)	0.38085 (7)	0.0122 (2)	
C14A	0.19800 (15)	0.31049 (10)	0.48803 (8)	0.0168 (2)	
C15A	0.09808 (16)	0.15392 (11)	0.59046 (10)	0.0240 (3)	
H15A	0.1383	0.0784	0.6190	0.029*	
H15B	0.0522	0.1506	0.5418	0.029*	
C16A	−0.02486 (18)	0.20524 (12)	0.66008 (10)	0.0256 (3)	
H16A	−0.1042	0.1596	0.6867	0.038*	
H16B	−0.0702	0.2780	0.6310	0.038*	
H16C	0.0209	0.2112	0.7072	0.038*	
C17A	0.84315 (13)	0.53960 (9)	0.34180 (8)	0.0138 (2)	
H17A	0.8501	0.5088	0.4052	0.017*	
H17B	0.8806	0.6101	0.3221	0.017*	
C18A	0.94678 (13)	0.46049 (10)	0.28325 (8)	0.0145 (2)	
H18A	1.0479	0.4387	0.2990	0.017*	
H18B	0.9011	0.3939	0.2975	0.017*	
C19A	0.75854 (17)	1.07453 (11)	0.07806 (10)	0.0230 (3)	
H19A	0.8692	1.0722	0.0635	0.035*	
H19B	0.7082	1.1312	0.1116	0.035*	
H19C	0.7223	1.0909	0.0225	0.035*	
O1B	0.62907 (11)	0.10394 (8)	0.35690 (7)	0.0212 (2)	
O2B	0.38151 (10)	0.10387 (7)	0.35342 (6)	0.01744 (18)	
O3B	0.29192 (11)	0.76162 (7)	0.15387 (6)	0.01769 (18)	
N1B	0.71022 (11)	0.49118 (8)	0.12914 (6)	0.01173 (18)	
N2B	0.48235 (11)	0.56326 (8)	0.08697 (6)	0.01134 (18)	
C1B	0.62243 (14)	0.78327 (9)	0.00981 (8)	0.0148 (2)	
H1BA	0.5224	0.7990	0.0449	0.018*	
C2B	0.69291 (15)	0.86841 (10)	−0.05266 (9)	0.0184 (2)	
H2BA	0.6389	0.9410	−0.0587	0.022*	
C3B	0.84224 (15)	0.84821 (11)	−0.10662 (8)	0.0196 (2)	
C4B	0.92369 (15)	0.73979 (11)	−0.09289 (9)	0.0192 (2)	
H4BA	1.0255	0.7250	−0.1260	0.023*	
C5B	0.85548 (14)	0.65327 (10)	−0.03060 (8)	0.0158 (2)	
H5BA	0.9119	0.5814	−0.0224	0.019*	
C6B	0.70215 (13)	0.67365 (9)	0.01996 (7)	0.0123 (2)	
C7B	0.63190 (12)	0.57798 (9)	0.08024 (7)	0.01131 (19)	
C8B	0.60714 (12)	0.41630 (9)	0.17018 (7)	0.01138 (19)	
C9B	0.62468 (13)	0.31246 (9)	0.22990 (7)	0.0124 (2)	
H9BA	0.7173	0.2821	0.2493	0.015*	
C10B	0.49860 (13)	0.25547 (9)	0.25962 (7)	0.0128 (2)	
C11B	0.35857 (13)	0.29980 (9)	0.22970 (8)	0.0138 (2)	
H11B	0.2779	0.2586	0.2496	0.017*	
C12B	0.33878 (13)	0.40355 (9)	0.17128 (8)	0.0130 (2)	
H12B	0.2465	0.4337	0.1515	0.016*	
C13B	0.46462 (12)	0.46028 (9)	0.14371 (7)	0.01133 (19)	
C14B	0.51372 (13)	0.14745 (9)	0.32729 (8)	0.0141 (2)	
C15B	0.38024 (15)	0.00252 (10)	0.42410 (9)	0.0191 (2)	
H15C	0.3812	0.0174	0.4819	0.023*	
H15D	0.4716	−0.0518	0.4078	0.023*	
C16B	0.23432 (17)	−0.04128 (12)	0.43262 (10)	0.0253 (3)	
H16D	0.2266	−0.1057	0.4822	0.038*	
H16E	0.2383	−0.0611	0.3766	0.038*	
H16F	0.1451	0.0150	0.4445	0.038*	
C17B	0.35660 (13)	0.63748 (9)	0.04793 (8)	0.0135 (2)	
H17C	0.3082	0.5961	0.0222	0.016*	
H17D	0.3990	0.6956	−0.0014	0.016*	
C18B	0.23360 (13)	0.69020 (10)	0.11993 (8)	0.0159 (2)	
H18C	0.1464	0.7322	0.0932	0.019*	
H18D	0.1953	0.6318	0.1706	0.019*	
C19B	0.9105 (2)	0.94054 (13)	−0.17975 (10)	0.0301 (3)	
H19D	1.0220	0.9215	−0.1936	0.045*	
H19E	0.8801	1.0081	−0.1582	0.045*	
H19F	0.8726	0.9508	−0.2340	0.045*	
H1OA	0.875 (3)	0.5068 (18)	0.1692 (14)	0.039 (6)*	
H1OB	0.328 (3)	0.7248 (19)	0.1973 (15)	0.035 (5)*	

### Atomic displacement parameters (Å^2^)

**Table:**

	*U*^11^	*U*^22^	*U*^33^	*U*^12^	*U*^13^	*U*^23^
O1A	0.0201 (5)	0.0313 (5)	0.0292 (5)	−0.0108 (4)	−0.0094 (4)	0.0060 (4)
O2A	0.0170 (4)	0.0157 (4)	0.0259 (5)	−0.0055 (3)	−0.0013 (3)	0.0032 (3)
O3A	0.0102 (4)	0.0206 (4)	0.0134 (4)	−0.0030 (3)	−0.0024 (3)	−0.0036 (3)
N1A	0.0129 (4)	0.0138 (4)	0.0123 (4)	−0.0020 (3)	−0.0024 (3)	−0.0008 (3)
N2A	0.0108 (4)	0.0132 (4)	0.0113 (4)	−0.0018 (3)	−0.0021 (3)	−0.0011 (3)
C1A	0.0133 (5)	0.0140 (5)	0.0164 (5)	−0.0012 (4)	−0.0024 (4)	−0.0016 (4)
C2A	0.0141 (5)	0.0154 (5)	0.0187 (5)	−0.0029 (4)	−0.0031 (4)	0.0004 (4)
C3A	0.0182 (5)	0.0140 (5)	0.0173 (5)	−0.0029 (4)	−0.0062 (4)	−0.0014 (4)
C4A	0.0183 (5)	0.0131 (5)	0.0187 (5)	0.0011 (4)	−0.0047 (4)	−0.0037 (4)
C5A	0.0153 (5)	0.0151 (5)	0.0167 (5)	−0.0008 (4)	−0.0027 (4)	−0.0041 (4)
C6A	0.0129 (5)	0.0132 (5)	0.0124 (5)	−0.0016 (4)	−0.0028 (4)	−0.0018 (4)
C7A	0.0120 (5)	0.0128 (5)	0.0120 (4)	−0.0009 (4)	−0.0020 (4)	−0.0024 (4)
C8A	0.0122 (5)	0.0130 (5)	0.0106 (4)	−0.0016 (4)	−0.0017 (4)	−0.0020 (4)
C9A	0.0117 (5)	0.0155 (5)	0.0134 (5)	−0.0020 (4)	−0.0020 (4)	−0.0026 (4)
C10A	0.0148 (5)	0.0145 (5)	0.0121 (5)	−0.0035 (4)	−0.0009 (4)	−0.0027 (4)
C11A	0.0150 (5)	0.0144 (5)	0.0132 (5)	−0.0023 (4)	−0.0020 (4)	−0.0011 (4)
C12A	0.0127 (5)	0.0145 (5)	0.0131 (5)	−0.0008 (4)	−0.0029 (4)	−0.0012 (4)
C13A	0.0119 (5)	0.0130 (5)	0.0103 (4)	−0.0017 (4)	−0.0008 (3)	−0.0023 (4)
C14A	0.0177 (5)	0.0167 (5)	0.0149 (5)	−0.0049 (4)	−0.0017 (4)	−0.0021 (4)
C15A	0.0205 (6)	0.0164 (5)	0.0307 (7)	−0.0084 (4)	0.0015 (5)	−0.0008 (5)
C16A	0.0277 (7)	0.0268 (6)	0.0219 (6)	−0.0118 (5)	−0.0004 (5)	−0.0035 (5)
C17A	0.0117 (5)	0.0163 (5)	0.0137 (5)	−0.0020 (4)	−0.0038 (4)	−0.0031 (4)
C18A	0.0116 (5)	0.0164 (5)	0.0142 (5)	−0.0001 (4)	−0.0041 (4)	−0.0021 (4)
C19A	0.0263 (7)	0.0144 (5)	0.0257 (6)	−0.0042 (5)	−0.0074 (5)	0.0022 (5)
O1B	0.0168 (4)	0.0184 (4)	0.0249 (5)	−0.0033 (3)	−0.0078 (4)	0.0044 (3)
O2B	0.0155 (4)	0.0163 (4)	0.0181 (4)	−0.0053 (3)	−0.0037 (3)	0.0021 (3)
O3B	0.0189 (4)	0.0150 (4)	0.0177 (4)	−0.0003 (3)	−0.0036 (3)	−0.0039 (3)
N1B	0.0096 (4)	0.0128 (4)	0.0121 (4)	−0.0017 (3)	−0.0029 (3)	−0.0013 (3)
N2B	0.0092 (4)	0.0110 (4)	0.0126 (4)	−0.0003 (3)	−0.0035 (3)	−0.0009 (3)
C1B	0.0140 (5)	0.0136 (5)	0.0165 (5)	−0.0008 (4)	−0.0053 (4)	−0.0023 (4)
C2B	0.0203 (6)	0.0148 (5)	0.0206 (6)	−0.0038 (4)	−0.0089 (4)	0.0003 (4)
C3B	0.0213 (6)	0.0207 (6)	0.0174 (5)	−0.0093 (4)	−0.0068 (4)	0.0021 (4)
C4B	0.0142 (5)	0.0233 (6)	0.0176 (5)	−0.0059 (4)	−0.0003 (4)	−0.0016 (4)
C5B	0.0126 (5)	0.0171 (5)	0.0161 (5)	−0.0023 (4)	−0.0023 (4)	−0.0021 (4)
C6B	0.0110 (5)	0.0138 (5)	0.0117 (4)	−0.0021 (4)	−0.0038 (4)	−0.0011 (4)
C7B	0.0091 (4)	0.0129 (5)	0.0116 (4)	−0.0013 (3)	−0.0022 (3)	−0.0028 (4)
C8B	0.0092 (4)	0.0137 (5)	0.0111 (4)	−0.0015 (3)	−0.0020 (3)	−0.0031 (4)
C9B	0.0098 (4)	0.0145 (5)	0.0124 (5)	−0.0004 (3)	−0.0031 (4)	−0.0030 (4)
C10B	0.0121 (5)	0.0130 (5)	0.0121 (5)	−0.0013 (4)	−0.0020 (4)	−0.0024 (4)
C11B	0.0120 (5)	0.0145 (5)	0.0143 (5)	−0.0027 (4)	−0.0027 (4)	−0.0020 (4)
C12B	0.0100 (5)	0.0151 (5)	0.0137 (5)	−0.0019 (4)	−0.0034 (4)	−0.0027 (4)
C13B	0.0100 (4)	0.0123 (4)	0.0113 (4)	−0.0011 (3)	−0.0028 (3)	−0.0023 (4)
C14B	0.0135 (5)	0.0144 (5)	0.0137 (5)	−0.0027 (4)	−0.0021 (4)	−0.0025 (4)
C15B	0.0195 (6)	0.0163 (5)	0.0188 (5)	−0.0061 (4)	−0.0048 (4)	0.0033 (4)
C16B	0.0210 (6)	0.0237 (6)	0.0298 (7)	−0.0086 (5)	−0.0022 (5)	−0.0032 (5)
C17B	0.0108 (5)	0.0134 (5)	0.0157 (5)	0.0008 (4)	−0.0063 (4)	−0.0013 (4)
C18B	0.0107 (5)	0.0154 (5)	0.0196 (5)	0.0004 (4)	−0.0032 (4)	−0.0028 (4)
C19B	0.0342 (8)	0.0284 (7)	0.0255 (7)	−0.0173 (6)	−0.0068 (6)	0.0079 (5)

### Geometric parameters (Å, °)

**Table:**

O1A—C14A	1.2051 (17)	O1B—C14B	1.2082 (15)
O2A—C14A	1.3422 (15)	O2B—C14B	1.3483 (14)
O2A—C15A	1.4591 (15)	O2B—C15B	1.4454 (14)
O3A—C18A	1.4160 (14)	O3B—C18B	1.4166 (15)
O3A—H1OA	0.96 (2)	O3B—H1OB	0.81 (2)
N1A—C7A	1.3302 (15)	N1B—C7B	1.3291 (14)
N1A—C8A	1.3868 (14)	N1B—C8B	1.3902 (13)
N2A—C7A	1.3752 (15)	N2B—C13B	1.3787 (14)
N2A—C13A	1.3846 (14)	N2B—C7B	1.3793 (14)
N2A—C17A	1.4586 (15)	N2B—C17B	1.4545 (14)
C1A—C2A	1.3899 (16)	C1B—C2B	1.3875 (16)
C1A—C6A	1.3959 (16)	C1B—C6B	1.3993 (16)
C1A—H1AA	0.9300	C1B—H1BA	0.9300
C2A—C3A	1.3896 (17)	C2B—C3B	1.3928 (18)
C2A—H2AA	0.9300	C2B—H2BA	0.9300
C3A—C4A	1.3978 (17)	C3B—C4B	1.3919 (19)
C3A—C19A	1.5038 (17)	C3B—C19B	1.5072 (18)
C4A—C5A	1.3875 (16)	C4B—C5B	1.3896 (16)
C4A—H4AA	0.9300	C4B—H4BA	0.9300
C5A—C6A	1.3985 (16)	C5B—C6B	1.4017 (16)
C5A—H5AA	0.9300	C5B—H5BA	0.9300
C6A—C7A	1.4729 (15)	C6B—C7B	1.4718 (15)
C8A—C9A	1.3923 (15)	C8B—C9B	1.3919 (15)
C8A—C13A	1.4037 (16)	C8B—C13B	1.4039 (15)
C9A—C10A	1.3906 (16)	C9B—C10B	1.3954 (15)
C9A—H9AA	0.9300	C9B—H9BA	0.9300
C10A—C11A	1.4078 (17)	C10B—C11B	1.4082 (16)
C10A—C14A	1.4889 (16)	C10B—C14B	1.4858 (16)
C11A—C12A	1.3879 (15)	C11B—C12B	1.3840 (16)
C11A—H11A	0.9300	C11B—H11B	0.9300
C12A—C13A	1.3945 (16)	C12B—C13B	1.3929 (14)
C12A—H12A	0.9300	C12B—H12B	0.9300
C15A—C16A	1.495 (2)	C15B—C16B	1.5060 (18)
C15A—H15A	0.9700	C15B—H15C	0.9700
C15A—H15B	0.9700	C15B—H15D	0.9700
C16A—H16A	0.9600	C16B—H16D	0.9600
C16A—H16B	0.9600	C16B—H16E	0.9600
C16A—H16C	0.9600	C16B—H16F	0.9600
C17A—C18A	1.5230 (16)	C17B—C18B	1.5272 (16)
C17A—H17A	0.9700	C17B—H17C	0.9700
C17A—H17B	0.9700	C17B—H17D	0.9700
C18A—H18A	0.9700	C18B—H18C	0.9700
C18A—H18B	0.9700	C18B—H18D	0.9700
C19A—H19A	0.9600	C19B—H19D	0.9600
C19A—H19B	0.9600	C19B—H19E	0.9600
C19A—H19C	0.9600	C19B—H19F	0.9600
			
C14A—O2A—C15A	115.51 (11)	C14B—O2B—C15B	116.22 (10)
C18A—O3A—H1OA	108.5 (13)	C18B—O3B—H1OB	108.2 (15)
C7A—N1A—C8A	105.16 (10)	C7B—N1B—C8B	105.51 (9)
C7A—N2A—C13A	106.47 (9)	C13B—N2B—C7B	107.03 (9)
C7A—N2A—C17A	130.53 (9)	C13B—N2B—C17B	122.50 (9)
C13A—N2A—C17A	123.00 (10)	C7B—N2B—C17B	130.43 (10)
C2A—C1A—C6A	119.88 (11)	C2B—C1B—C6B	119.85 (11)
C2A—C1A—H1AA	120.1	C2B—C1B—H1BA	120.1
C6A—C1A—H1AA	120.1	C6B—C1B—H1BA	120.1
C3A—C2A—C1A	121.93 (11)	C1B—C2B—C3B	121.82 (12)
C3A—C2A—H2AA	119.0	C1B—C2B—H2BA	119.1
C1A—C2A—H2AA	119.0	C3B—C2B—H2BA	119.1
C2A—C3A—C4A	117.82 (11)	C4B—C3B—C2B	117.94 (11)
C2A—C3A—C19A	121.07 (11)	C4B—C3B—C19B	121.33 (12)
C4A—C3A—C19A	121.05 (11)	C2B—C3B—C19B	120.70 (13)
C5A—C4A—C3A	120.93 (11)	C5B—C4B—C3B	121.13 (11)
C5A—C4A—H4AA	119.5	C5B—C4B—H4BA	119.4
C3A—C4A—H4AA	119.5	C3B—C4B—H4BA	119.4
C4A—C5A—C6A	120.73 (11)	C4B—C5B—C6B	120.40 (11)
C4A—C5A—H5AA	119.6	C4B—C5B—H5BA	119.8
C6A—C5A—H5AA	119.6	C6B—C5B—H5BA	119.8
C1A—C6A—C5A	118.69 (10)	C1B—C6B—C5B	118.70 (10)
C1A—C6A—C7A	124.55 (10)	C1B—C6B—C7B	123.14 (10)
C5A—C6A—C7A	116.72 (10)	C5B—C6B—C7B	118.15 (10)
N1A—C7A—N2A	112.70 (10)	N1B—C7B—N2B	112.04 (9)
N1A—C7A—C6A	121.20 (10)	N1B—C7B—C6B	123.42 (10)
N2A—C7A—C6A	125.93 (10)	N2B—C7B—C6B	124.34 (10)
N1A—C8A—C9A	129.96 (11)	N1B—C8B—C9B	130.89 (10)
N1A—C8A—C13A	109.82 (10)	N1B—C8B—C13B	109.62 (10)
C9A—C8A—C13A	120.21 (10)	C9B—C8B—C13B	119.49 (10)
C10A—C9A—C8A	117.82 (11)	C8B—C9B—C10B	117.73 (10)
C10A—C9A—H9AA	121.1	C8B—C9B—H9BA	121.1
C8A—C9A—H9AA	121.1	C10B—C9B—H9BA	121.1
C9A—C10A—C11A	121.28 (11)	C9B—C10B—C11B	121.69 (10)
C9A—C10A—C14A	117.03 (11)	C9B—C10B—C14B	117.90 (10)
C11A—C10A—C14A	121.70 (11)	C11B—C10B—C14B	120.37 (10)
C12A—C11A—C10A	121.52 (11)	C12B—C11B—C10B	121.18 (10)
C12A—C11A—H11A	119.2	C12B—C11B—H11B	119.4
C10A—C11A—H11A	119.2	C10B—C11B—H11B	119.4
C11A—C12A—C13A	116.56 (11)	C11B—C12B—C13B	116.38 (10)
C11A—C12A—H12A	121.7	C11B—C12B—H12B	121.8
C13A—C12A—H12A	121.7	C13B—C12B—H12B	121.8
N2A—C13A—C12A	131.53 (11)	N2B—C13B—C12B	130.72 (10)
N2A—C13A—C8A	105.85 (10)	N2B—C13B—C8B	105.80 (9)
C12A—C13A—C8A	122.58 (10)	C12B—C13B—C8B	123.46 (10)
O1A—C14A—O2A	123.30 (11)	O1B—C14B—O2B	123.54 (11)
O1A—C14A—C10A	124.42 (12)	O1B—C14B—C10B	124.61 (11)
O2A—C14A—C10A	112.29 (11)	O2B—C14B—C10B	111.84 (10)
O2A—C15A—C16A	111.79 (11)	O2B—C15B—C16B	107.68 (11)
O2A—C15A—H15A	109.3	O2B—C15B—H15C	110.2
C16A—C15A—H15A	109.3	C16B—C15B—H15C	110.2
O2A—C15A—H15B	109.3	O2B—C15B—H15D	110.2
C16A—C15A—H15B	109.3	C16B—C15B—H15D	110.2
H15A—C15A—H15B	107.9	H15C—C15B—H15D	108.5
C15A—C16A—H16A	109.5	C15B—C16B—H16D	109.5
C15A—C16A—H16B	109.5	C15B—C16B—H16E	109.5
H16A—C16A—H16B	109.5	H16D—C16B—H16E	109.5
C15A—C16A—H16C	109.5	C15B—C16B—H16F	109.5
H16A—C16A—H16C	109.5	H16D—C16B—H16F	109.5
H16B—C16A—H16C	109.5	H16E—C16B—H16F	109.5
N2A—C17A—C18A	112.15 (9)	N2B—C17B—C18B	111.50 (9)
N2A—C17A—H17A	109.2	N2B—C17B—H17C	109.3
C18A—C17A—H17A	109.2	C18B—C17B—H17C	109.3
N2A—C17A—H17B	109.2	N2B—C17B—H17D	109.3
C18A—C17A—H17B	109.2	C18B—C17B—H17D	109.3
H17A—C17A—H17B	107.9	H17C—C17B—H17D	108.0
O3A—C18A—C17A	113.28 (9)	O3B—C18B—C17B	112.66 (9)
O3A—C18A—H18A	108.9	O3B—C18B—H18C	109.1
C17A—C18A—H18A	108.9	C17B—C18B—H18C	109.1
O3A—C18A—H18B	108.9	O3B—C18B—H18D	109.1
C17A—C18A—H18B	108.9	C17B—C18B—H18D	109.1
H18A—C18A—H18B	107.7	H18C—C18B—H18D	107.8
C3A—C19A—H19A	109.5	C3B—C19B—H19D	109.5
C3A—C19A—H19B	109.5	C3B—C19B—H19E	109.5
H19A—C19A—H19B	109.5	H19D—C19B—H19E	109.5
C3A—C19A—H19C	109.5	C3B—C19B—H19F	109.5
H19A—C19A—H19C	109.5	H19D—C19B—H19F	109.5
H19B—C19A—H19C	109.5	H19E—C19B—H19F	109.5
			
C6A—C1A—C2A—C3A	−0.49 (18)	C6B—C1B—C2B—C3B	0.21 (18)
C1A—C2A—C3A—C4A	1.04 (18)	C1B—C2B—C3B—C4B	−3.33 (18)
C1A—C2A—C3A—C19A	−176.41 (11)	C1B—C2B—C3B—C19B	174.55 (12)
C2A—C3A—C4A—C5A	−1.06 (18)	C2B—C3B—C4B—C5B	3.20 (19)
C19A—C3A—C4A—C5A	176.39 (11)	C19B—C3B—C4B—C5B	−174.67 (12)
C3A—C4A—C5A—C6A	0.55 (18)	C3B—C4B—C5B—C6B	0.03 (19)
C2A—C1A—C6A—C5A	−0.06 (17)	C2B—C1B—C6B—C5B	3.06 (17)
C2A—C1A—C6A—C7A	177.64 (11)	C2B—C1B—C6B—C7B	−175.93 (11)
C4A—C5A—C6A—C1A	0.03 (17)	C4B—C5B—C6B—C1B	−3.18 (17)
C4A—C5A—C6A—C7A	−177.85 (11)	C4B—C5B—C6B—C7B	175.86 (11)
C8A—N1A—C7A—N2A	0.84 (12)	C8B—N1B—C7B—N2B	−0.29 (12)
C8A—N1A—C7A—C6A	−174.66 (9)	C8B—N1B—C7B—C6B	−175.30 (10)
C13A—N2A—C7A—N1A	−1.15 (12)	C13B—N2B—C7B—N1B	−0.12 (12)
C17A—N2A—C7A—N1A	178.46 (10)	C17B—N2B—C7B—N1B	177.66 (10)
C13A—N2A—C7A—C6A	174.10 (10)	C13B—N2B—C7B—C6B	174.83 (10)
C17A—N2A—C7A—C6A	−6.29 (18)	C17B—N2B—C7B—C6B	−7.39 (17)
C1A—C6A—C7A—N1A	−144.30 (12)	C1B—C6B—C7B—N1B	−142.71 (11)
C5A—C6A—C7A—N1A	33.45 (15)	C5B—C6B—C7B—N1B	38.29 (15)
C1A—C6A—C7A—N2A	40.83 (17)	C1B—C6B—C7B—N2B	42.90 (16)
C5A—C6A—C7A—N2A	−141.43 (11)	C5B—C6B—C7B—N2B	−136.10 (11)
C7A—N1A—C8A—C9A	178.75 (11)	C7B—N1B—C8B—C9B	−178.72 (11)
C7A—N1A—C8A—C13A	−0.21 (12)	C7B—N1B—C8B—C13B	0.59 (12)
N1A—C8A—C9A—C10A	−178.00 (10)	N1B—C8B—C9B—C10B	−179.58 (10)
C13A—C8A—C9A—C10A	0.88 (16)	C13B—C8B—C9B—C10B	1.16 (15)
C8A—C9A—C10A—C11A	0.54 (16)	C8B—C9B—C10B—C11B	0.93 (16)
C8A—C9A—C10A—C14A	−179.57 (10)	C8B—C9B—C10B—C14B	−176.73 (9)
C9A—C10A—C11A—C12A	−1.38 (17)	C9B—C10B—C11B—C12B	−1.80 (17)
C14A—C10A—C11A—C12A	178.74 (10)	C14B—C10B—C11B—C12B	175.80 (10)
C10A—C11A—C12A—C13A	0.71 (16)	C10B—C11B—C12B—C13B	0.48 (16)
C7A—N2A—C13A—C12A	−176.79 (11)	C7B—N2B—C13B—C12B	−178.09 (11)
C17A—N2A—C13A—C12A	3.56 (18)	C17B—N2B—C13B—C12B	3.91 (17)
C7A—N2A—C13A—C8A	0.93 (11)	C7B—N2B—C13B—C8B	0.47 (11)
C17A—N2A—C13A—C8A	−178.72 (9)	C17B—N2B—C13B—C8B	−177.53 (9)
C11A—C12A—C13A—N2A	178.14 (11)	C11B—C12B—C13B—N2B	−179.96 (11)
C11A—C12A—C13A—C8A	0.75 (16)	C11B—C12B—C13B—C8B	1.69 (16)
N1A—C8A—C13A—N2A	−0.46 (12)	N1B—C8B—C13B—N2B	−0.67 (12)
C9A—C8A—C13A—N2A	−179.55 (9)	C9B—C8B—C13B—N2B	178.74 (9)
N1A—C8A—C13A—C12A	177.51 (10)	N1B—C8B—C13B—C12B	178.03 (9)
C9A—C8A—C13A—C12A	−1.57 (17)	C9B—C8B—C13B—C12B	−2.56 (16)
C15A—O2A—C14A—O1A	0.05 (18)	C15B—O2B—C14B—O1B	3.17 (17)
C15A—O2A—C14A—C10A	179.75 (10)	C15B—O2B—C14B—C10B	−175.76 (9)
C9A—C10A—C14A—O1A	4.82 (18)	C9B—C10B—C14B—O1B	−1.86 (17)
C11A—C10A—C14A—O1A	−175.29 (12)	C11B—C10B—C14B—O1B	−179.55 (11)
C9A—C10A—C14A—O2A	−174.87 (10)	C9B—C10B—C14B—O2B	177.06 (9)
C11A—C10A—C14A—O2A	5.02 (16)	C11B—C10B—C14B—O2B	−0.64 (15)
C14A—O2A—C15A—C16A	−77.41 (15)	C14B—O2B—C15B—C16B	−171.37 (10)
C7A—N2A—C17A—C18A	−102.64 (13)	C13B—N2B—C17B—C18B	73.62 (13)
C13A—N2A—C17A—C18A	76.92 (13)	C7B—N2B—C17B—C18B	−103.86 (13)
N2A—C17A—C18A—O3A	70.06 (12)	N2B—C17B—C18B—O3B	65.15 (12)

### Hydrogen-bond geometry (Å, °)

**Table:**

Cg1 is the centroid of the N1B/C7B/N2B/C8B/C13B 4,5-dihydro imidazole ring.

**Table:**

*D*—H···*A*	*D*—H	H···*A*	*D*···*A*	*D*—H···*A*
O3A—H1OA···N1B	0.96 (3)	1.83 (3)	2.7847 (14)	173 (2)
O3B—H1OB···N1A	0.81 (2)	2.08 (2)	2.8859 (15)	172 (3)
C1A—H1AA···O3A	0.93	2.37	3.2473 (16)	156
C1B—H1BA···O3B	0.93	2.36	3.2331 (16)	157
C12B—H12B···O3A^i^	0.93	2.45	3.2788 (16)	149
C15B—H15C···O1B^ii^	0.97	2.55	3.2889 (17)	133
C18A—H18A···O1A^iii^	0.97	2.58	3.1928 (16)	121
C17B—H17C···Cg1^iv^	0.97	2.70	3.4194 (13)	131

Symmetry codes: (i) *x*−1, *y*, *z*; (ii) −*x*+1, −*y*, −*z*+1; (iii) *x*+1, *y*, *z*; (iv) −*x*+1, −*y*+1, −*z*.
